# Anorexia nervosa in later life: a case report and review of the literature

**DOI:** 10.1192/j.eurpsy.2026.11364

**Published:** 2026-07-31

**Authors:** F. Zaouali, I. Anes

**Affiliations:** 1 Emergency department and mobile emergency and resuscitation service, Tahar Sfar University Hospital, Mahdia; 2 Outpatient psychiatric consultation, Hadj Ali Soua Regional Hospital, Ksar Hellal, Monastir, Tunisia

## Abstract

**Introduction:**

Anorexia nervosa (AN) is classically considered a disorder of adolescence and young adulthood. However, late-onset cases, defined as onset after age 30, are increasingly recognized, although rare and often underdiagnosed. AN in later life may present with atypical clinical features, frequently misattributed to aging or comorbid conditions.

**Objectives:**

To highlight the clinical and psychopathological characteristics of AN with onset in later life, identify common triggers and diagnostic challenges, and review available evidence on treatment outcomes.

**Methods:**

This study presents a case of late-onset AN in a 65-year-old woman and reviews the current literature on this uncommon presentation. A narrative review was conducted using PubMed and PsycINFO. Keywords included “anorexia nervosa”, “late onset”, “older adults”, and “eating disorders in the elderly”. Articles published in English or French over the past 30 years were screened. Inclusion criteria covered case reports, reviews, and clinical studies involving patients ≥40 years old at the time of AN onset. Out of 67 identified articles, 24 were included in the final analysis.

**Results:**

A 65-year-old female presented to the psychiatric consultation with progressive weight loss (BMI decline from 20.5 to 16.4 kg/m^2^ in 8 months), fatigue, and restrictive eating behaviors without purging or significant body image distortion. She reported a strong fear of becoming dependent and a desire to “stay in control” after her husband’s death. Medical evaluation excluded organic causes, and a diagnosis of late-onset restrictive anorexia nervosa was established. Management included hospitalization for nutritional support, pharmacologic treatment of comorbid depression, and psychotherapy focused on grief and autonomy, resulting in partial weight recovery and improved insight. The literature review revealed that late-onset AN predominantly affects women and is often triggered by life transitions such as bereavement, retirement, or social isolation. Unlike younger patients, older adults may not exhibit classical body image disturbances but instead experience fears related to aging, illness, or dependency. Diagnostic delays are common, and the condition is often misattributed to depression or somatic illness. Prognosis tends to be poorer due to medical comorbidities and under-recognition. Nonetheless, multidisciplinary interventions involving psychiatry, nutrition, and psychosocial support have demonstrated positive outcomes when implemented early.

**Conclusions:**

Anorexia nervosa can occur de novo in later life and should not be dismissed as a rare anomaly. Clinicians must maintain a high index of suspicion, especially in elderly patients with weight loss and food avoidance behaviors. Early identification and integrated management, including psychiatric, nutritional, and psychosocial interventions, are critical to reducing morbidity and improving prognosis in this vulnerable population.

**Disclosure of Interest:**

None Declared

